# Untangling the Ties Between Social Cognition and Body Motion: Gender Impact

**DOI:** 10.3389/fpsyg.2020.00128

**Published:** 2020-02-06

**Authors:** Sara Isernia, Alexander N. Sokolov, Andreas J. Fallgatter, Marina A. Pavlova

**Affiliations:** ^1^Department of Psychiatry and Psychotherapy, Medical School and University Hospital, Eberhard Karls University of Tübingen, Tübingen, Germany; ^2^Department of Psychology, Università Cattolica del Sacro Cuore, Milan, Italy; ^3^CADITeR, IRCCS Fondazione Don Carlo Gnocchi ONLUS, Milan, Italy

**Keywords:** biological motion, visual social cognition, gender, emotion, body language reading

## Abstract

We proved the viability of the general hypothesis that biological motion (BM) processing serves as a hallmark of social cognition. We assumed that BM processing and inferring emotions through BM (body language reading) are firmly linked and examined whether this tie is gender-specific. Healthy females and males completed two tasks with the same set of point-light BM displays portraying angry and neutral locomotion of female and male actors. For one task, perceivers had to indicate actor gender, while for the other, they had to infer the emotional content of locomotion. Thus, with identical visual input, we directed task demands either to BM processing or inferring of emotion. This design allows straight comparison between sensitivity to BM and recognition of emotions conveyed by the same BM. In addition, perceivers were administered a set of photographs from the Reading the Mind in the Eyes Test (RMET), with which they identified either emotional state or actor gender. Although there were no gender differences in performance on BM tasks, a tight link occurred between recognition accuracy of emotions and gender through BM in males. In females only, body language reading (both accuracy and response time) was associated with performance on the RMET. The outcome underscores gender-specific modes in visual social cognition and triggers investigation of body language reading in a wide range of neuropsychiatric disorders.

## Introduction

**Figure 1:**
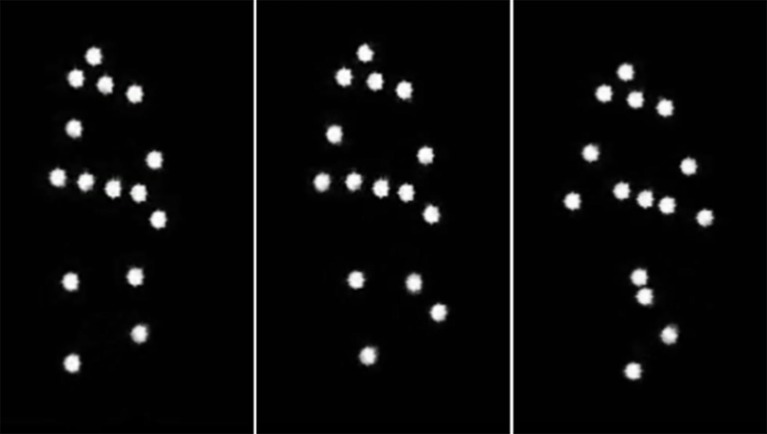
Illustration of point-light biological motion. Three consequent static frames exemplifying human walking as a set of dots placed on the main joints and head of an invisible actor body. A walker is seen facing left in intermediate position between the frontal and sagittal view.

Body language reading is an essential ability for efficient daily interpersonal exchanges and adaptive behavior. Another benefit of body language reading is that, although verbal information flow is believed to be easily kept under control, body movement often reveals our true feelings and dispositions. Typically developing (TD) individuals are proficient in inferring emotions and intentions of others represented by biological motion (BM) in *point-light* displays minimizing the availability of other cues (such as body shape or outfit) and, thereby, isolating information conveyed by BM solely (Figure 1) (e.g., Dittrich et al., 1996; Pollick et al., 2001; Atkinson et al., 2004; Heberlein et al., 2004; Clarke et al., 2005; Manera et al., 2010; Alaerts et al., 2011; Sokolov et al., 2011; Krüger et al., 2013; Actis-Grosso et al., 2015; Vaskinn et al., 2016). Perceivers can judge emotional content of dance, represented by a few moving dots located on a dancer’s body, with anger being the most reliably identified emotion (Dittrich et al., 1996). Inferring emotions from BM is fairly robust across cultures (Parkinson et al., 2017), and it remains rather accurate over age: only the recognition of sadness (but not angry or happy displays that are more exaggerated) at short durations is lower in the elderly (Spencer et al., 2016).

It has been argued that social cognitive abilities (i.e., abilities to perceive and understand emotional states, drives, and intentions of others) and BM processing are tightly linked and, therefore, performance on *socially neutral* tasks (such as detection of camouflaged BM, facing detection, or discrimination between canonical and scrambled BM) may serve as a hallmark of social cognition (Pavlova, 2012): individuals with neurodevelopmental and psychiatric conditions (such as autism spectrum disorders (ASD), Williams-Beuren syndrome, and Down syndrome) and survivors of premature birth who exhibit aberrant BM processing, have compromised daily social perception and possess lower social competence. In agreement with this assumption, newborn human infants (and newly hatched chicks) appear to be predisposed to BM, though such predispositions are impaired in newborns at high risk of autism (Bidet-Ildei et al., 2014; Di Giorgio et al., 2016, 2017).

In the non-clinical adult population, a possible intrinsic link between the ability to perceive BM and a person’s social capabilities appears to be in line with visual psychophysics. Emotional valence of BM affects the sensitivity to point-light gait masked by an additional set of dots taken from the same walker, with highest sensitivity (but also greatest response bias), to angry and lowest sensitivity, to neutral walking (Chouchourelou et al., 2006). The sensitivity to slightly camouflaged BM is related to both anger and happiness (Ikeda and Watanabe, 2009). Happiness superiority effect in BM processing is also affirmed: BM detection within noise is not only facilitated by an actor’s happiness, but happiness is easier to recognize than angry and neutral BM (Lee and Kim, 2017). The ability to reveal the identity of point-light dancers and expression intensity correlates with self-reported empathy (Sevdalis and Keller, 2011). Emotion recognition through BM is related not only to more basic capability for discrimination between canonical and scrambled BM, but also to performance on the Reading the Mind in the Eyes Test, RMET (Alaerts et al., 2011). Empathy, performance on both the RMET and Cambridge Face Memory Test, and autism quotient are all positively linked in TD individuals with efficient BM processing (such as facing detection) (Miller and Saygin, 2013). In children aged 7–12 years, BM facing detection is already associated with mindreading in eyes (Rice et al., 2016). Alexithymia (the inability to identify and describe emotions in the self) scores in TD individuals correlate with confidence in rating the emotion valence of point-light BM displays (Lorey et al., 2012). BM processing (decoding of gender) is affected by gender stereotyping elicited by depicted emotion: angry throwing of a ball is often judged to be performed by men, whereas sad throwing is judged to be performed by women (Johnson et al., 2011). Inferring emotions through BM is modulated by administration of the neuropeptide oxytocin known to facilitate social cognition (Bernaerts et al., 2016; Wynn et al., 2019). Moreover, smelling steroids (either androstadienone or estratetraenol) makes observers to estimate the emotional state of a point-light walker of the opposite sex as happier and more relaxed (Ye et al., 2019).

Some aspects of BM processing and/or body language reading are aberrant in psychiatric, neurological, psychosomatic, and neurodevelopmental disorders (for reviews, see Pavlova, 2012; Pavlova, 2017a,b; Okruszek et al., 2018). Most importantly, the visual sensitivity to BM is inversely linked to the severity of these disorders, e.g., as measured by the autistic diagnostic observation schedule (ADOS) in ASD (Blake et al., 2003), or to autism traits in TD (Koldewyn et al., 2010). For ASD and TD individuals pooled together, both BM processing and emotion recognition are related to social responsiveness scores (Nackaerts et al., 2012). In schizophrenia (SZ), deficient BM is connected to aberrant social cognition (Okruszek and Pilecka, 2017; Okruszek, 2018). For instance, deficient BM detection (discrimination between such actions as climbing a stair and scrambled displays) is linked to lower social competence (Kim et al., 2005). In SZ, a positive correlation is reported between BM processing (detection of facing direction of masked walkers) and the empathy index (Matsumoto et al., 2015). Poorer emotion recognition is associated with impaired self-reported social functional capacity, community outcome (such as lifetime relationship status and independent living) and, in particular, in individuals who committed homicide, with a tendency to under-mentalize (Engelstad et al., 2017, 2018a,b; Egeland et al., 2019).

In a number of tasks examining BM processing, gender/sex differences are reported (Pavlova, 2017a,b). TD adult females are more accurate in BM recognition of actions (such as jumping on the spot) and faster in discriminating between emotional and neutral BM (Alaerts et al., 2011). Yet gender differences in body language reading appear to be modulated by the type of portrayed emotion and actor gender (Sokolov et al., 2011; Krüger et al., 2013). TD females are reported to be more accurate in body language reading through full-light body motion (Strauss et al., 2015). Brain imaging points to sex differences in neural circuits underpinning BM processing (Anderson et al., 2013; Pavlova et al., 2015). Sex differences in BM processing are also reported in other species (Regolin et al., 2000; Brown et al., 2010). This points to their fundamental character. Gender (socio-cultural aspects) and sex (neurobiological aspects) impacts can be of substantial value, not only for better conceptualization of social cognition but also for understanding neuropsychiatric conditions, most of which are gender/sex specific (Pavlova, 2012, 2017a,b).

Some previous studies on social cognition through BM possess methodological limitations: (1) BM tests are often based on videotapes of only one (either female or male) or two (female and male) performers. For example, many studies on emotion recognition from BM in psychiatric populations use videos of only one actor [e.g., *EmoBio test* first introduced by Heberlein et al. (2004); see also Okruszek et al., 2018]. (2) Socially neutral BM processing and inferring social information from point-light displays are often assessed with different sets (or types) of displays and experimental procedures. (3) Unbalanced design is used with samples of TD individuals and patients that are not properly matched in respect to gender (e.g., patients of one gender are compared with TD individuals of both genders) and/or differ in sample size (sample of TD individuals is twice or even larger than patient sample). If samples contain many more individuals of one gender and/or more TD participants than patients, comparisons between groups may lead to paradoxical statistical outcomes. These issues can preclude proper generalization of findings.

Here we proved the viability of the assumption that BM processing is firmly linked with expressive body language reading. Bearing in mind the occurrence of gender-specific modes in both BM processing and social cognition, we examined whether this bond is gender-specific. For this purpose, TD females and males completed two tasks with the same set of point-light BM displays portraying angry and neutral locomotion of female and male actors. On one task, perceivers had to indicate an actor’s gender, whereas on the other, the emotional content of locomotion. Thus, with identical visual input, we directed task demands either to BM processing or emotion recognition. The primary benefit of this design is that it allows comparison between BM processing and inferring emotions conveyed by the same BM. In addition, in a separate session, perceivers were administered a set of photographs from the RMET for identifying either an emotional state or actor gender.

## Materials and Methods

### Participants

Forty participants (20 females and 20 males, aged 19–39 years; students of the University of Tübingen Medical School) were enrolled in the study. No age difference occurred between them: males were aged 26.5 years [median (Mdn), 95% confidence interval, CI from 24.43 to 30.67], and females were aged 25 years [Mdn, 95% CI from 23.23 to 28.27 (Mann-Whitney test, *U* = 171.5, *p* = 0.439, n.s.)]. As performance on the RMET (German version, for details, see section below) requires language command of high proficiency, German as a native language served as an inclusion criterion. All observers had normal or corrected-to-normal vision. None had head injuries or a history of neuropsychiatric disorders (including ASD, SZ, and depression), or regular drug intake (medication). They were run individually and were naïve as to the purpose of the study. None had previous experience with such displays and tasks. The study was conducted in line with the Declaration of Helsinki and was approved by the local Ethics Committee at the University of Tübingen Medical School. Informed written consent was obtained from all participants. Participation was voluntary, and the data were processed anonymously.

### Biological Motion: Stimuli, Tasks, and Procedure

Participants were presented with a set of point-light black-and-white animations portraying human locomotion. Display production is described in detail elsewhere (Krüger et al., 2013). The displays were built up by using the Motion Capture Library. In brief, recording was performed using a 3D position measurement system at a rate of 60 Hz (Optotrak, Northern Digital Inc., Waterloo, ON, Canada). The matrix data for each frame were processed with MATLAB (The Mathworks Inc., Natick, MA, USA) into a video sequence. Each display consisted of 15 white dots visible against a black background (Figure 1). The dots were placed on the shoulder, elbow, and wrist of each arm; on the hip, knee, and ankle of each leg; and on the head, neck, and pelvis of a human body. As we intended to make tasks demanding and supposed more pronounced effects with brief stimulus duration, each movie lasted for 2 s which corresponded to one walking cycle consisting of two steps. During locomotion, a walker was seen facing right in an intermediate position of 45° between the frontal and sagittal view. As sagittal view is often considered neutral in respect to possible social interactions and the frontal view is reported to elicit ambiguous (facing backward or toward an observer) and often gender-dependent impressions of locomotion direction (Pollick et al., 2005; Brooks et al., 2008; Schouten et al., 2010, 2011), the intermediate trajectory of locomotion was used. For creation of left faced stimuli, we rotated videos 90° horizontally. The walking figure was positioned with the pelvis fixed to the middle of the screen. Female and male actors walked either with angry or neutral affective expression. For avoiding variability in emotion portrayal, sets of neutral and angry stimuli were created from the same actors. The videos of six (three female/three male) actors facing either right or left were presented in three separate runs with a short break between them. In total, each experimental session consisted of a set of 144 trials [6 actors (3 female/3 male) × 2 emotions (neutral/angry) × 2 facing directions (left/right) × 6 (2 repetitions of each stimulus in each run × 3 runs) trials. During an inter-stimulus interval (after stimulus offset and till onset of the next stimulus right after participant’s response), a white fixation cross was displayed in the center of the screen for 6 s. If a participant failed to respond within this period, the next trial automatically started. Participants were asked to respond after each stimulus offset.

With the same set of stimuli, in a two-alternative forced choice (2AFC) paradigm, participants performed two different tasks indicating by pressing one of two respective keys either actor gender (female/male) or emotion (neutral/angry). By contrast with emotion task, performance on gender task is based on revealing biomechanical characteristics of locomotion (Kozlowski and Cutting, 1977; Barclay et al., 1978; Cutting et al., 1978; Pollick et al., 2005). The order of tasks (gender/emotion recognition) was counterbalanced between participants. Using identical visual input (the same set of displays) in the same sample of participants, we varied task demands re-directing the task either to BM processing or to bodily emotion recognition. The whole experimental session (consisting of two tasks) took about 20–25 min per participant. No immediate feedback was given regarding performance. The main advantage of this experimental design is that it allows comparison between sensitivity to BM and recognition of emotions conveyed by the same BM.

### Reading Mind in the Eyes Test and Gender Recognition Task

After completion of both BM tasks, a computer version of the RMET was additionally administered to participants. This test is described in detail elsewhere (Baron-Cohen et al., 2001). In brief, participants were shown a set of 36 black-and-white photographs of female and male eyes along with a corresponding face part expressing a certain emotional or affective state. On each trial, they had to choose among four alternative descriptions (adjectives) simultaneously presented on the screen including the correct one corresponding with the picture. Participants were instructed to be as fast as possible. Each correct response was scored 1 for a total score range of 0–36. This standardized test is considered one of the most commonly used tasks assessing affective theory of mind (Baglio and Marchetti, 2016). The test was administered in a computerized form by 2019 Qualtrics^®^; https://www.qualtrics.com/de/ (Qualtrics International Inc.; Provo, Utah, U. S. A.). In the other session, with the same set of 36 photographs, participants completed a gender recognition task from RMET (RMET_G). The RMET was used primarily for proving whether affect recognition through body motion and eye expressions are linked to each other. Gender recognition on the RMET_G task was used as a control.

### Data Analysis

Data analysis was performed by using Statistical Package for Social Science (SPSS version 24, IBM Corporation; Armonk, New York, U.S.A.) and JMP Software (version 13; SAS Institute; Cary, North Carolina, U.S.A.). All data were tested for normality of distribution by Shapiro-Wilk test with subsequent uses of either parametric (for normally distributed data sets) or, otherwise, non-parametric statistics.

## Results

### Biological Motion Tasks: Emotion and Gender Recognition

In accordance with our assumption that BM processing is firmly linked to expressive body language reading, our data analysis was primarily focused on associations between performance on the emotion recognition task (BME) and the gender recognition task (BMG); the outcome of the analysis of variance (ANOVA) is reported for completeness.

Individual rates of correct responses on both BM tasks were submitted to a mixed model 2 × 2 × 2 × 2 repeated-measures omnibus ANOVA with within-subject factors Task (gender/emotion recognition), Actor Gender (female/male), and Emotion (angry/neutral), and a between-subject factor Observer Gender (female/male). The outcome revealed main effects of Task [*F*(1,38) = 24.46, *p* < 0.001] with higher accuracy on revealing emotions than gender, Actor Gender [*F*(1,38) = 37.62, *p* < 0.001] with higher accuracy in recognition of movies of male than female actors, and Emotion [*F*(1,38) = 64.71; *p* < 0.001] with better performance for neutral than angry displays on both tasks together. The main effect of Observer Gender only tended to be significant [*F*(1,38) = 3.41; *p* = 0.066] with a non-significant interaction between Observer Gender and Task [*F*(1,38) = 0.99, *p* = 0.321, n.s.]. All interactions are summarized in Supplementary Table S1. *Post hoc* analysis indicated a lack of gender differences in accuracy on both BM tasks [BME: *t*(38) = 0.92, *p* = 0.365, n.s., and BMG: *t*(38) = 1.39, *p* = 0.173, n.s., two-tailed]. Similarly, no gender differences in response time were found [BME: *U* = 197, *p* = 0.935, n.s.; BMG: *t*(38) = 1.46, *p* = 0.153; n.s., two-tailed].

**Figure 2:**
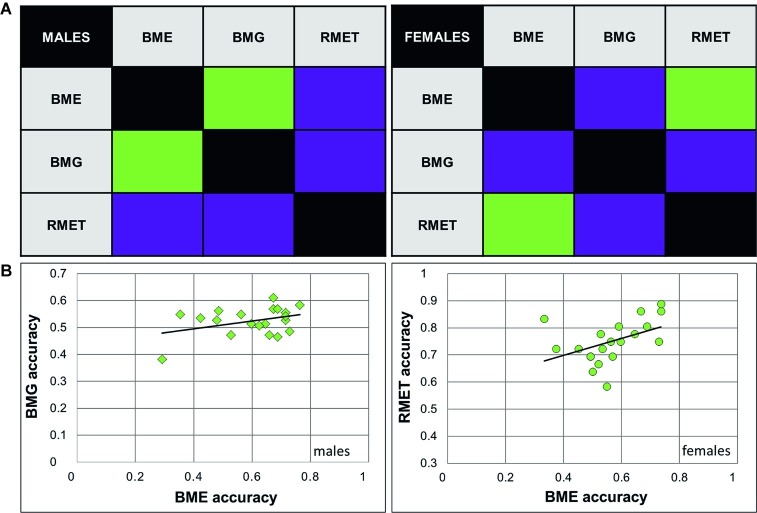
Relationship between accuracy of emotion and gender recognition through biological motion, and performance on the Reading Mind in the Eyes Test for female and male observers. **(A)** Correlation matrices between accuracy of performance (correct response rate) on emotion (BME) and gender (BMG) recognition through biological motion (BM), and the Reading Mind in the Eyes Test (RMET). Significant correlations (Pearson product-moment correlation; *p* < 0.05) are color-coded by green, non-significant correlation by violet. **(B)** Correlations between BMG and BME accuracy in males (left panel, diamonds), and between the RMET and BME accuracy in females (right panel, circles) were significant.

**Figure 3:**
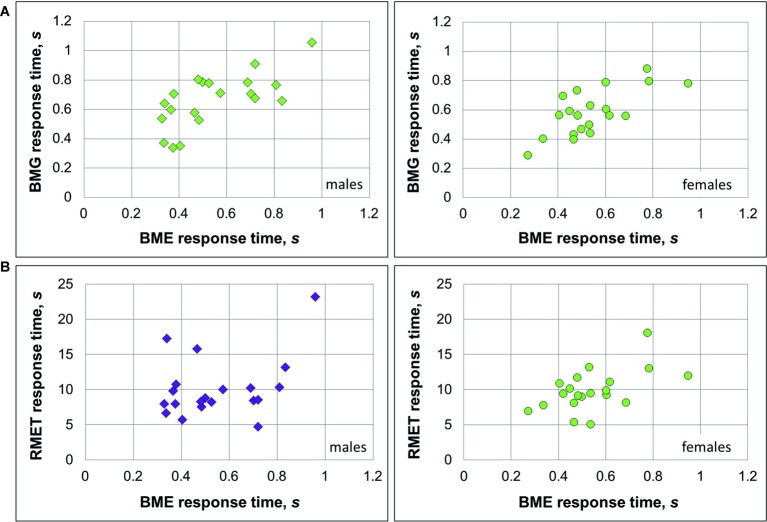
Relationship between response time on emotion and gender recognition through biological motion, and the Reading Mind in the Eyes Test (RMET) for female and male observers. **(A)** Both in males (left panel, diamonds) and females (right panel, circles) BMG and BME response time were significantly linked to each other. **(B)** In males (left panel), response time on the BME task and RMET were not associated with each other, whereas in females (right panel) this association was significant. Significant correlations (Spearman’s *ρ*; *p* < 0.05) are color-coded by green, non-significant correlations by violet.

As we expected to find a positive link between recognition of emotions (BME task) and gender (BMG task) through BM, correlation analysis was conducted on performance accuracy and response time separately for females and males. In males, accuracy of emotion and gender recognition through BM was positively linked with each other [Pearson product-moment correlation, *r*(18) = 0.38, *p* = 0.049; Figure 2A], whereas no such association was found in females [*r*(18) = 0.032, *p* = 0.447, n.s., both one-tailed]. Response time of correct responses between the BME and BMG tasks positively correlated with each other in both gender groups [males: Spearman’s *ρ*(18) = 0.633, *p* = 0.002; females: *ρ*(18) = 0.568, *p* = 0.005, both one-tailed; Figure 3A].

### Relation Between Inferring Emotion Through Biological Motion and Reading the Mind in the Eyes Test

We administered a set of stimuli from the RMET primarily for addressing the issue of whether performance on two visual social cognition tasks, namely, revealing eyes expressions (RMET) and emotion recognition through BM (BME) are connected to each other. Gender recognition with the RMET set of stimuli (RMET_G task) served as a control.

As expected from previous work (Kirkland et al., 2013), females were more proficient on the RMET with greater recognition accuracy [*t*(38) = 1.73, *p* = 0.046, one-tailed, effect size Cohen’s *d* = 0.56]. Notably, we found that females tended to surpass males in recognition of female images [*t*(38) = 1.97, *p* = 0.056, two-tailed], with no gender difference in recognition of male images [*U* = 185, *p* = 0.677, n.s., two-tailed]. No gender difference on the RMET task was found in response time (*U* = 183, *p* = 0.323, n.s.). No gender difference in recognition accuracy occurred on the RMET_G task (*U* = 187.5, *p* = 0.724, n.s.), presumably because the task turned to be far too easy to perform. There was also no gender difference in response time on this task (*U* = 199, *p* = 0.978, n.s.). No correlation occurred between recognition accuracy on the RMET and RMET_G task [males: Spearman’s *ρ*(18) = −0.161, *p* = 0.497; females: *ρ*(18) = 0.17, *p* = 0.473, n.s.].

Based on earlier work (Alaerts et al., 2011; Miller and Saygin, 2013), we expected to find a positive link between accuracy on the BME and RMET tasks. Yet, in males, the correlation between recognition accuracy on these tasks turned to be non-significant [*r*(18) = 0.186, *p* = 0.216, n.s], whereas in females accuracy of BME and RMET positively correlated with each other [*r*(18) = 0.445, *p* = 0.025, one-tailed; Figure 2B]. Similarly, response time on the BME task and RMET correlated with each other in females [*ρ*(18) = 0.483, *p* = 0.016; Figure 3B, right panel], but not in males [*ρ*(18) = 0.287, *p* = 0.11, n.s., one-tailed; Figure 3B, left panel].

## Discussion

The present study was aimed at the proof of concept in accord with which body motion perception and visual social cognition are intimately tied (Pavlova, 2012). Keeping in mind experimental evidence for gender-specific modes in both visual social cognition and BM processing, we focused on the gender specificity of this link. The findings revealed that: (1) A tight link occurred between the accuracy of gender and emotion recognition through BM in males, though there were no gender differences in performance on both BM tasks. Independent of observers’ gender, response time on emotion and gender recognition through BM correlated with each other. (2) In females only, body language reading (both accuracy and response time) was associated with mindreading through eyes.

The outcome provides further support for the general concept according with which BM processing serves a hallmark of social cognition (Pavlova, 2012). Previous research already pointed to the link between BM processing and social cognition: individuals with aberrant BM processing are also compromised on daily-life social cognition possessing lower social competence, empathy, and face recognition capabilities (Sevdalis and Keller, 2011; Miller and Saygin, 2013). In this study, we tried to untangle the ties between BM processing and body language reading by using identical visual input and re-directing task demands either to BM processing [gender decoding that is based on revealing biomechanical characteristics of locomotion (Kozlowski and Cutting, 1977; Barclay et al., 1978; Cutting et al., 1978; Pollick et al., 2005)] or to emotion recognition. For the first time, we uncovered the gender specificity of these ties. It appears that males heavily rely upon common mechanisms underpinning gender and emotion recognition through BM, whereas in females, this tie is not so pronounced: only response time but not accuracy of gender and emotion recognition are positively linked to each other. This outcome appears to dovetail with recent reports indicating that females and males tend to use different types of information during BM processing and gender recognition in point-light displays: females rely on form and motion cues together, whereas males use motion cues solely (Hiris et al., 2018). This is also in line with recent findings on gender recognition in human infants aged 4–18 months: in a habituation paradigm, boys more easily differentiate the gender of a point-light walker, presumably possessing higher sensitivity to motion parameters (Murray et al., 2018; Tsang et al., 2018). Yet adaptation effects in point-light BM gender recognition indicate that this process is rather unlikely to be based on extracting low-level perceptual features (Jordan et al., 2006). In accord with this, in SZ individuals, both emotion and gender recognition of avatars correlate with social functioning: emotion recognition correlates with the level of social engagement and interpersonal communication, whereas gender recognition is linked with independence in daily life (Peterman et al., 2014). Future brain imaging research will help to clarify where and how gender and emotion recognition through BM talk to each other in the brain.

By contrast, females likely bank on tightly interconnected general mechanisms of social cognition for emotion recognition through BM and mindreading through eyes. In males, the link in performance between these tasks is absent. At first glance, bearing in mind previous reports (Alaerts et al., 2011; Miller and Saygin, 2013) on the association between emotion recognition through point-light BM and eye expressions on the RMET, gender specificity of this linkage (occurrence of this link in females only) in the present study appears rather startling. Yet in these earlier studies, samples of participants contained predominately females.

In agreement with previous work (Kirkland et al., 2013) that points to female superiority on the RMET (independent of cultural differences), females tended to outperform males at judging mental states expressed by eyes. Yet there were no gender differences on the emotion through BM task. Brain imaging work on BM processing and inferring social interaction through Heider-and-Simmel-like animations suggests the existence of gender-specific modes in processing of socially relevant information even in the absence of behavioral differences: gender-related dimorphism in the neural circuits may prevent behavioral differences if they are maladaptive, and thereby promote proper behavioral response (Pavlova et al., 2010, 2015). Similarly, implementing different behavioral strategies by females and males may have contributed to the lack of gender differences in performance on BM tasks in the present study.

The present study was conducted in the student sample that affords group homogeneity. Although such a population is commonly used in the field, this may represent a limitation in terms of the outcome generalizability. However, since the study was focused on the association between performances on the tests, one would expect that, in general population, perceivers who are proficient on one task may be expected to be more proficient on the other and vice versa.

Gender specificity of the link between BM processing and visual social cognition may be of value for better understanding a wide range of psychiatric, neurologic, neurodevelopmental, and psychosomatic conditions. Some aspects of BM processing are atypical in ASD (e.g., Klin et al., 2009; Nackaerts et al., 2012; Jack et al., 2017), schizophrenia (e.g., Kim et al., 2011; Hastings et al., 2013; Spencer et al., 2013; Hashimoto et al., 2014; Vaskinn et al., 2016, 2018; Engelstad et al., 2017, 2018a,b; Okruszek et al., 2018) and schizotypal personality disorder (Hur et al., 2016), bipolar disorders (Vaskinn et al., 2017), attention deficit hyperactivity disorder (ADHD) (Kröger et al., 2014), anxiety disorders and in individuals with elevated anxiety (van de Cruys et al., 2013; Heenan and Troje, 2015), obsessive compulsive disorders (Kim et al., 2008), and unipolar depression (Loi et al., 2013; Kaletsch et al., 2014). Deficits are also reported in individuals who were born preterm and suffer congenital brain lesions (Pavlova and Krägeloh-Mann, 2013), Alzheimer’s (Henry et al., 2012; Insch et al., 2015) and Parkinson’s diseases (Cao et al., 2015; Jaywant et al., 2016a,b; Kloeters et al., 2017), epilepsy (Bala et al., 2018), and eating disorders such as anorexia nervosa and bulimia (Zucker et al., 2013; Lang et al., 2015; Dapelo et al., 2017). Most of these disorders that are characterized by aberrant social cognition display a skewed sex ratio: females and males are affected differently in terms of clinical picture, prevalence, and severity (Pavlova, 2012, 2017a,b).

BM processing relies on a large-scale neural network (Grosbras et al., 2012; Engell and McCarthy, 2013; Pavlova et al., 2017). For understanding proper functioning of this network and especially its pathology, one has to consider dynamic changes in brain activation unfolding over time (Pavlova, 2017a,b). Recently, whole-head ultrahigh field 9.4 T functional magnetic resonance imaging (fMRI), along with temporal analysis of blood-oxygen-level-dependent (BOLD) responses, revealed distinct large-scale ensembles of regions playing in unison during different stages of BM processing (Pavlova et al., 2017). An integrative analysis of structural and effective brain connectivity sheds light on architecture and functional principles of the BM circuitry, which is organized in a parallel rather than hierarchical manner (Sokolov et al., 2018). The hub of this circuitry lies in the right posterior superior temporal sulcus, STS (Grossman and Blake, 2002; Beauchamp et al., 2003; Gobbini et al., 2007; Kaiser et al., 2010; Herrington et al., 2011; Dasgupta et al., 2017), where this network likely communicates with the social brain, the neural circuits underwriting our ability for perception and understanding of drives, intentions, and emotions of others. The visual sensitivity to BM is best predicted by functional communication (effective connectivity) and presence of white-matter pathways between the right STS and fusiform gyrus (Sokolov et al., 2018).

Research on the brain networks dedicated to affective body language reading in normalcy and pathology is extremely sparse (Heberlein et al., 2004; Atkinson et al., 2012; Jastorff et al., 2015; Mazzoni et al., 2017; He et al., 2018). This work emphasizes the key role of the STS and fusiform face area in inferring emotions of point-light agents and avatars (e.g., Goldberg et al., 2015; Vonck et al., 2015). In a nutshell, it appears that BM processing engages a specialized neural network with hubs in the several areas of the brain including the right temporal cortex and fusiform gyrus, where this circuitry topographically overlaps and communicates with the social brain. Specifically tailored brain imaging is required to clarify to what extent visual processing of BM and expressive body language reading share topographically and dynamically overlapping neural networks. This work will contribute to better understanding of neurodevelopmental, psychiatric, neurological, and psychosomatic disorders related to social cognition.

## Resume

The present study was aimed at providing a proof of concept that BM perception and visual social cognition are intimately tied (Pavlova, 2012). Here, we focused on the gender specificity of this bond. By using identical visual input and re-directing task demands either to BM processing or emotion recognition, we cautiously untangled the ties between BM processing and body language reading. The findings revealed that (1) although there were no gender differences in performance on both BM tasks, a tight link occurred between accuracy of gender and emotion recognition through BM in males. (2) In females only, body language reading is linked with mindreading through eyes. The outcome points to gender-specific modes in visual social cognition and fosters investigation of body language reading in a wide range of neuropsychiatric disorders.

## Data Availability Statement

The raw data will be made available by the authors, without undue reservation, to any qualified researcher.

## Ethics Statement

The studies involving human participants were reviewed and approved by the Ethical Committee of the University of Tübingen Medical School. The participants provided their written informed consent to participate in this study.

## Author Contributions

MP, SI, and AF conceived and designed the study experiments. SI performed the experiments. MP, SI, and AS analyzed the data. MP and AF contributed reagents, materials, and analysis tools. SI and MP wrote the paper. MP supervised the whole project. All co-authors contributed to the writing of the manuscript.

### Conflict of Interest

The authors declare that the research was conducted in the absence of any commercial or financial relationships that could be construed as a potential conflict of interest.
