# Supplementary material for: Untangling the Ties Between Social Cognition and Body Motion: Gender Impact
Source: Front Psychol. 2020 Feb 6;11:128. doi: 10.3389/fpsyg.2020.00128 (PMC7016199; doi:10.3389/fpsyg.2020.00128)
Supplement: Supplementary file 1 [file Table_1.docx]

**Supplementary Table S1.** The outcome of ANOVA for accuracy of emotion and gender recognition through biological motion (BM).

|  | *df* | SSq | *F* | *p* |
| --- | --- | --- | --- | --- |
| OBSERVER GENDER | 1 | 0.049 | 3.409 | 0.066 |
| ACTOR GENDER | 1 | 0.544 | 37.616 | <.0001 |
| EMOTION | 1 | 0.936 | 64.709 | <.0001 |
| TASK | 1 | 0,354 | 24.456 | <.0001 |
| OBSERVER GENDER * EMOTION | 1 | 0.002 | 0.104 | 0.747 |
| OBSERVER GENDER * ACTOR GENDER | 1 | 0.071 | 4.875 | 0.028 |
| OBSERVER GENDER * TASK | 1 | 0.014 | 0.989 | 0.321 |
| ACTOR GENDER * EMOTION | 1 | 0.092 | 6.340 | 0.012 |
| ACTOR GENDER * TASK | 1 | 0.245 | 16.966 | <0.001 |
| EMOTION * TASK | 1 | 0.814 | 56.278 | <0.001 |

**Two-way interactions** (for main effects, see article text)

1. Interaction of factors Observer Gender and Actor Gender (F(1;38) = 4.88, p = 0.028) indicated that difference in recognition accuracy of movies portraying male and female actors was greater in female than male observers. Pairwise comparisons (Tukey honestly significant difference (HSD) tests, two-tailed, corrected for multiple comparisons) revealed that for both (emotion and gender recognition) tasks together, (*i*) male observers better recognized movies of male than female actors (t(266) = 2.78, p = 0.03), the same held true for female observers (t(266) = 5.9, p < 0.001); (*iii*) males better recognized movies of male actors (t(266) = 5.64, p < 0.001) and female actors (t(266) = 2.87, p = 0.023) than females recognized movies of female actors, and (*iii*) females better recognized movies of male actors than males recognized movies of female actors (t(266) = 3.03, p = 0.014).

2. Interaction of factors Actor Gender and Emotion (F(1;38) = 6.34, p = 0.012) indicated that difference in recognition accuracy between movies depicting neutral and angry emotions was greater for movies of female than male actors. For both tasks together, (*i*) movies of male actors were better recognizable when they expressed neutral than angry locomotion (t(266) = 3.91, p < 0.001), the same held true for movies of female actors (t(266) = 7.47, p < 0.001); (*ii*) with angry locomotion only, movies of male actors were better recognizable than movies of female actors (t(266) = 6.12, p < 0.001) and (*iii*) movies of male actors with neutral locomotion were recognized better than movies of females with angry locomotion (t(266) = 10.02, p < 0.001).

3. Interaction of factors Actor Gender and Task (F(1;38) = 16.97, p < 0.001) indicated that difference in recognition accuracy between emotion and gender tasks was greater for movies of female compared to male actors. Independent of expressed emotion, (*i*) on gender recognition through BM task, movies of male actors were better recognizable than female actors (t(266) = 7.25, p < 0.001), (*ii*) movies of female actors were better recognizable on emotion compared to gender through BM task (t(266) = 6.41, p < 0.001), and (*iii*) movies of male actors in emotion task were better recognizable than movies of females actors in gender task (t(266) = 7.83, p < 0.001).

4. Interaction of factors Emotion and Task (F(1;38) = 56.28, p < 0.001) indicated that difference in recognition accuracy between emotion and gender tasks was greater for movies representing neutral compared to angry locomotion. Independent of actor gender, (*i*) on emotion task, movies with neutral locomotion were better recognizable than movies with angry locomotion (t(266) = 10.99, p < 0.001), (*ii*) movies with neutral locomotion on emotion task were better recognizable than movies with angry locomotion on gender task (t(266) = 9.18, p < 0.001), and (*iii*) movies with neutral locomotion were better recognizable on emotion than on gender task (t(266) = 8.80, p < 0.001).

All other two-way interactions and pairwise comparisons failed to attain significance. Three-way interactions are not reported due to the known uncertainty of their interpretation.
